# Deep blue autofluorescence reflects the oxidation state of human transthyretin

**DOI:** 10.1016/j.redox.2022.102434

**Published:** 2022-08-09

**Authors:** Elżbieta Wieczorek, Zofia Wygralak, Sylwia Kędracka-Krok, Patrycja Bezara, Dominika Bystranowska, Piotr Dobryszycki, Andrzej Ożyhar

**Affiliations:** a Department of Biochemistry, Molecular Biology and Biotechnology, Faculty of Chemistry, Wrocław University of Science and Technology, Wybrzeże Wyspiańskiego 27, 50-370, Wrocław, Poland; b Department of Physical Biochemistry, Faculty of Biochemistry, Biophysics and Biotechnology, Jagiellonian University, Kraków, Poland

**Keywords:** Multiple sclerosis, Amyloid, Aging

## Abstract

Human transthyretin (TTR) is a tetrameric protein transporting thyroid hormones and retinol. TTR is a neuroprotective factor and sensor of oxidative stress which stability is diminished due to mutations and aging, leading to amyloid deposition. Adverse environmental conditions, such as redox and metal ion imbalances, induce destabilization of the TTR structure. We have previously shown that the stability of TTR was disturbed by Ca^2+^ and other factors, including DTT, and led to the formation of an intrinsic fluorophore(s) emitting blue light, termed deep blue autofluorescence (dbAF). Here, we show that the redox state of TTR affects the formation dynamics and properties of dbAF. Free thiols lead to highly unstable subpopulations of TTR and the frequent ocurrence of dbAF. Oxidative conditions counteracted the destabilizing effects of free thiols to some extent. However, strong oxidative conditions led to modifications of TTR, which altered the stability of TTR and resulted in unique dbAF spectra. Riboflavin and/or riboflavin photoproducts bound to TTR and crosslinked TTR subunits. Riboflavin-sensitized photooxidation increased TTR unfolding, while photooxidation, either in the absence or presence of riboflavin, increased proteolysis and resulted in multiple oxidative modifications and dityrosine formation in TTR molecules. Therefore, oxidation can switch the role of TTR from a protective to pathogenic factor.

## Graphical abstract

**Figure:**
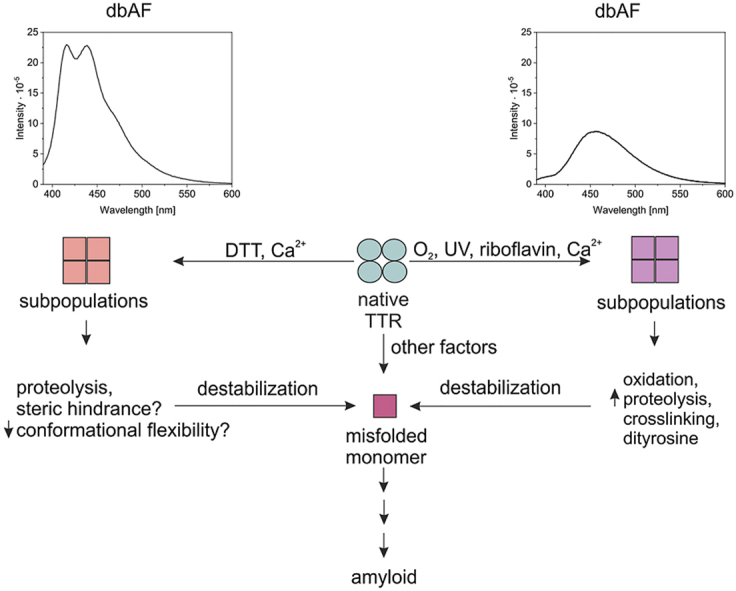

## 1 Introduction

Human transthyretin (UniProtKB, P02766) is a 55 kDa protein (Protparam, https://web.expasy.org/protparam/) composed of four 127 amino-acid-long monomers that form a central channel upon association. TTR is mainly synthesized in the liver and in epithelial cells of the choroid plexus and is released into plasma and cerebrospinal fluid (CSF), respectively [1]. TTR is also present (due to synthesis or internalization) in the pancreas, kidney, retinal pigment epithelium (RPE), interphotoreceptor matrix (IPM) and some cell types of the brain [1,2]. The central channel of TTR accommodates thyroid hormones but also binds, with different affinities, several natural ligands, including polyphenols, lipids, curcumin, norepinephrine oxidation products, lutein, quercetin, and many others [[3], [4], [5], [6], [7], [8]]. The transporting function of thyroid hormones via TTR in plasma is shared with albumin and thyroxine binding globulin. TTR also transports retinol due to association with the ligand-loaded retinol bindiing protein (RBP) [5]. TTR has neuroprotective functions and/or is a stress response factor in various adverse conditions, including ischemia and Alzheimer's disease [[1], [9], [10], [11], [12], [13]].

The structure of TTR has attracted much attention because, on the one hand, TTR is a highly kinetically stable protein [14], but on the other hand, when mutated or due to aging, it is prone to aggregation [[1], [15]]. Four TTR monomers (subunits A, B, C and D) associate into dimer of dimers. The interfaces between subunits A and B or C and D are engaged in the extensive network of hydrogen bonding (monomer-monomer interface), whereas the hydrophobic interactions between the subunits (A and C or B and D) are provided by loop regions (dimer-dimer interface) [16]. The β-sandwich structure of each TTR monomer is formed by two β-sheets composed of four β-strands (CBEF and DAGH) each. These antiparallel β-strands are connected by flexible loops that add some conformational freedom/flexibility to the rather rigid core [17,18]. One of the loops (EF loop) accommodates one short α-helix [16]. Tetramer dissociation and partial monomer unfolding have been found to initiate TTR aggregation [15,17]. The tetramers dissociate into dimers followed by rapid dissociation to monomers [19]. The conformational fluctuations disrupting the DAGH β-sheet lead to local unfolding of the monomer [20]. Nonnative TTR monomers aggregate into oligomers of diverse stoichiometry and structure followed by the formation of protofibrils and fibrils. Structural rearrangements occur throughout the whole multistep process of TTR aggregation into mature fibrils [21,22]. Low-populated cytotoxic transient intermediates of TTR escape from the ground state, and the dynamics of the structural fluctuation of TTR molecules have been found to underlie the aggregation process [17].

The factors responsible for destabilization of TTR structure increase the amyloid formation ability of TTR, which underlies diseases such as familial amyloidotic polyneuropathy (FAP), familial amyloidotic cardiomyopathy (FAC), senile systemic amyloidosis (SSA) and atherosclerosis, and cardiovascular and osteoarticular diseases [23,24]. The process of aggregation can be initiated by most known TTR mutations and by environmental factors, such as low pH (lysosomal compartments), high concentrations of ions and unfavorable/imbalanced redox conditions [15,24,25]. TTR undergoes metal ion-induced proteolytic fragmentation of the N-terminus around the Cys10-Pro11 peptide bond [26]. The amyloid deposits found in FAP patients (type B amyloid) contain the full length mutated TTR molecules; however, in the SSA and some FAP patients, the amyloid of type A was observed. Amyloid A, in addition to full-length wild-type or mutated TTR, also contains the *C*-terminal fragment lacking the 48 amino-acid-long N-terminus of TTR [[24], [27]]. The mechanism responsible for amyloid A formation is still unrecognized, although it has recently been proven that TTR is the substrate for plasmin. Plasmin cleavage results in TTR fragmentation corresponding to that found in amyloid A [28].

Another important factor that induces TTR destabilization is age-related oxidation [29]. The amino acid residues of TTR, which are particularly susceptible/vulnerable to oxidation and/or modification, are Cys10 and Met13. A single Cys10 is located in the vicinity of strand A and a single Met13 is located on strand A of each monomer of wild-type TTR. Due to its sensitivity to redox conditions, Cys10 of TTR was proposed to be a sensor of the redox state of the environment [30,31]. The extent and modification type of Cys10 are correlated with the pathogenic process and the severity of the disease [32]. In particular, the specific oxidative modification of TTR Cys10 residue in the CSF has been found to be associated with demyelinating diseases (multiple sclerosis and acute disseminated encephalomyelitis) [33].

Previously, we showed that the process of TTR destabilization was induced by Ca^2+^ (in the absence and presence of DTT) in the subpopulations of TTR and was accompanied by the formation of the new intrinsic fluorophore(s) dbAF [34]. dbAF formation is associated with structural arrangements that lower the energy barrier of fluorophore excitation and enable the emission of light in the blue range of the spectrum [35]. We intended to further investigate the influence of redox conditions (in the context of Ca^2+^) on the formation and dbAF properties of human TTR. The redox potential in the experiments was changed from more reducing to more oxidizing. We found that the formation dynamics of dbAF depend on the balance between reducing (free thiols) and oxidizing (irradiation, long incubation time, photosensitizer presence) conditions and that the obtained dbAF spectra differ. However, both conditions affected the fluorescence properties and stability and led to unstable subpopulations of TTR molecules. Riboflavin-enhanced photooxidation resulted in unfolding, dityrosine formation, crosslinking and enhanced proteolysis of TTR molecules. The addition of multiple oxygen atoms was found to accompany the photooxidation of TTR.

## 2 Materials and methods

### 2.1 Buffers

Predominantly and when not stated otherwise, 50 mM Tris, 100 mM NaCl, pH 7.3 (Tris buffer) was used for TTR samples. HEPES buffer (10 mM HEPES, 100 mM NaCl, pH 7.3) was used for irradiation experiments with riboflavin and mass spectrometry (MS). Both buffers were preincubated overnight with Chelex 100 resin (BioRad) to remove divalent ions according to the protocol supplied by the manufacturer.

### 2.2 Bacterial recombinant expression and purification of TTR

The expression and purification of recombinant TTR (possessing or devoid of the *C*-terminal histidine tag), which was cloned into the pGEX-2T vector, was performed according to Ref. [34]. Briefly, the expression of TTR fused to glutathione-S-transferase (GST) in the *E. coli* BL21 pLys cell line was induced with 1 mM isopropyl β-d-1-thiogalactopyranoside. The cells were cultured for 3 h at 37 °C, harvested by centrifugation, washed and suspended in phosphate-buffered saline (PBS) containing 1 mM DTT and frozen at −80 °C. Prior to purification, the cell suspension was thawed and lysed by pipetting. DNase I (10 μg/mL), RNase A (10 μg/mL) and phenylmethylsulfonyl fluoride (0.2 mg/mL) were added during cell lysis, and the soluble fraction was obtained by centrifugation. TTR was purified using a two-step protocol. First, the supernatant (containing fusion protein) was loaded on a glutathione-Sepharose column equilibrated with PBS, and contaminating proteins were washed out with PBS. Then, TTR was digested from the resin with thrombin (Calbiochem) at room temperature for up to 24 h. TTR-containing fractions were collected by washing the column with PBS, concentrated using a Centricon filter unit (Millipore), loaded onto a Superdex 200 column (30/300 GL, GE Healthcare) equilibrated with Tris buffer and separated using Akta Explorer (Amersham Biosciences).

### 2.3 Size exclusion chromatography and SDS–PAGE

Size exclusion chromatography was performed using a Superdex 75 Increase analytical column (10/300 GL, GE Healthcare, Chicago, IL, USA) preequilibrated with Tris buffer. Prior to separation, samples containing TTR, riboflavin, and CaCl_2_ in various combinations were irradiated at 23 °C for 30 min with light at an excitation wavelength of 445 nm using 2.0 nm slits. After irradiation, some samples were incubated overnight at 60 °C. Irradiated and nonirradiated samples were applied onto a column and analyzed in Tris buffer at a flow rate of 0.5 mL/min using Akta Explorer (GE Healthcare, Chicago, IL, USA).

For selected samples, 30 μL of the peak fractions was supplemented with four times concentrated sample buffer devoid or supplemented with 2.5% β-mercaptoethanol. Then, the samples were subjected to heat denaturation at 95 °C for 15 min or 20 min, except for the nonirradiated TTR sample, which was denatured at 85 °C for 30 min. All samples were loaded on 12% acrylamide gels containing SDS, run in a Laemmli buffer system and stained with Coomassie Brilliant Blue R250 and silver.

### 2.4 SDS-Trapping assay

Thermal unfolding of TTR was adopted from Ref. [14]. Samples, each containing one hundred micrograms of TTR in Tris buffer, were preincubated in the absence and presence of CaCl_2_ or riboflavin and subjected to irradiation at 23 °C for 30 min with light at an excitation wavelength of 445 nm and using 2.0 nm slits. One TTR sample (control) was not subjected to irradiation. Each sample was divided into nine portions containing 9 μg of TTR. At the same time, five times concentrated sample buffer (0.3 M Tris, 30% glycerol, 5% SDS, 0.05% bromophenol blue, pH 8.8**)** was preheated at 95 °C. Hot buffer was added to each 9 μg portion, and the tubes were immediately placed at 85 °C (*T*_*target*_) for thermal denaturation. The temperature for buffer preheating (*T*_*h*_) was calculated from the formula below.

$$T_{h}\left(K\right)\cdot mass\left(buffer\;SDS\;\right)+293.15\left(K\right)\cdot mass\left(protein\;sample\right)=T_{target}\cdot total\;mass$$

The samples were incubated at 85 °C, and unfolding was stopped after 0, 15, 30, 60, 120, 180, 300, 600 and 1200 s by placing the samples on ice for 3–5 s. The samples were centrifuged for 5 s at room temperature and immediately loaded on 12% acrylamide gels containing SDS. After separation, the gels were stained with Coomassie Brilliant Blue R250. After destaining, the intensities of the bands corresponding to the monomer and dimer were determined for each gel using Image Lab™ Software (BioRad, Hercules, CA, USA). The intensity of the dimer band at 0 s of thermal unfolding was set as the standard of quantity. The relative intensity of the monomer after time *i* of incubation was determined by calculating the R_u*i*_ parameter equal to (U_*i*_-U_0_)/(U_final_-U_0_), where U_*i*_, U_0_ and U_final_ are the intensities of the monomer bands at given times of 0 s and 1200 s, respectively. The MW was determined using the Thermo Scientific™ Pierce™ (Rockford, IL, USA) Unstained Protein Molecular Weight Marker.

### 2.5 Fluorescence measurements

***TTR spectra.*** The emission spectra of dbAF (from 375 to 600 nm), dityrosine (from 330 to 550 nm) and aromatic (from 300 to 400 nm) fluorophores were measured using excitation wavelengths of 360 nm, 315 nm and 275 nm, respectively. The fluorescence excitation spectra were collected in the ranges of 300–410 nm and 300–420 nm with the emission recorded at 438 nm and 455 nm, respectively. The spectra were recorded using a Fluorolog 3–21 Spex spectrofluorometer (Horiba). Fluorolog calibration was performed using the Ovalene standard. Measurements were performed in a 0.3 cm path length quartz cuvette (Hellma) using an integration time of 1 s and an increment of 1 nm. Slits with bandwidths of 3.0 nm were used for both the excitation and emission channels.

***TTR irradiation***. TTR samples were irradiated in a 0.3 cm path length quartz cuvette at 23 °C for 30 min using an excitation wavelength of 445 nm and slits of 2.0 nm in a Fluorolog spectrofluorometer (Horiba). All spectra were corrected by subtracting the spectra of the corresponding buffer (containing low molecular weight components) alone.

### 2.6 Mass spectrometry

TTR samples (35 μM monomer) in HEPES buffer were preincubated in the absence or presence of riboflavin (100 μM) and subjected to irradiation at 23 °C for 30 min with light at an excitation wavelength of 445 nm using 2.0 nm slits. The irradiated samples were incubated overnight at 60 °C and subjected to size-exclusion chromatography on a Superdex 75 Increase column. The peak protein fractions and samples of nonirradiated TTR (50 μM monomer) in HEPES buffer, which were preincubated in the absence and presence of 200 mM CaCl_2_, were subjected to MS. The protein samples were vacuum dried prior to MS analysis, resuspended in 2% acetonitrile with 0.05% TFA and centrifuged at 21,000×*g* for 15 min at 4 °C. Fifty nanograms of each protein sample was separated on a 15 cm × 75 μm Accucore™ 150-C4 column (particle size 2.6 μm, pore size 150 Å; Thermo Fisher Scientific, Waltham, MA, USA) with mobile phases of A (0.05% formic acid in water) and B (0.05% formic acid in acetonitrile). The proteins were eluted at a flow rate of 0.3 μL/min with an acetonitrile gradient that consisted of 2–25% B for 2 min, 25–60% B for 16 min, and 60–90% B for 2 min. The chromatograms were recorded, and MS spectra for the main protein peaks of the chromatograms were obtained using a MicrOTOF-QII mass spectrometer (Bruker, Billerica, MA, USA) equipped with a nanosprayer coupled with the UltiMate 3000 RSLCnano System (Dionex, Sunnyvale, CA, USA). The MW determination of the protein by a deconvolution multiply charged ion series was performed using Maximum Entropy software.

## 3 Results

### 3.1 The dynamics of dbAF fluorophore(s) formation in TTR are dependent on the redox potential of the environment

**Fig. 1:**
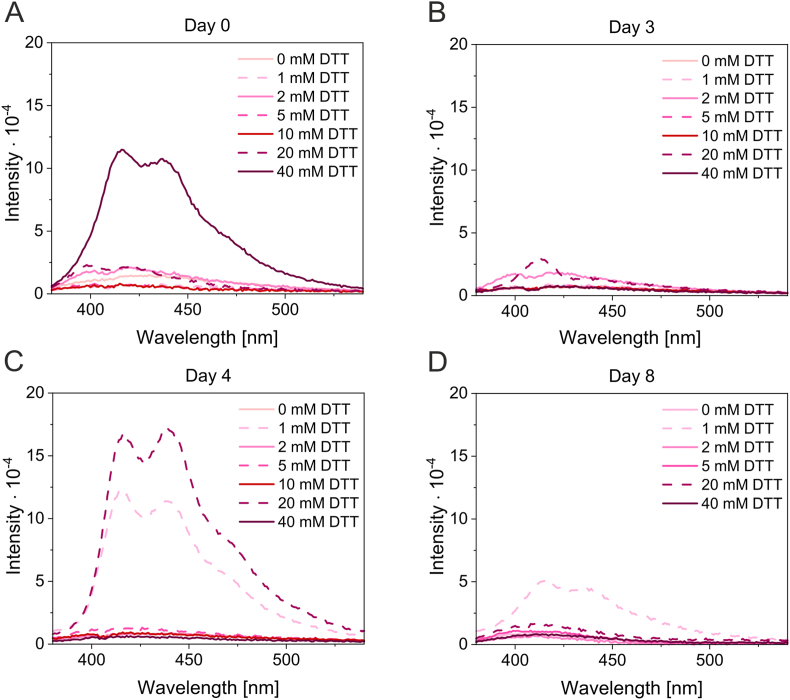
**Redox potential (different DTT concentrations) affects the dynamics of dbAF formation** Samples of TTR (2.7 μM) in Tris buffer containing 200 mM CaCl_2_ were supplemented with DTT to obtain the indicated final concentrations. After incubation at room temperature, the fluorescence emission spectra were recorded using an excitation wavelength of 360 nm. The spectra obtained for each concentration of DTT were superimposed for each incubation time, which is indicated at the top of each panel.

Unique dynamics of dbAF formation depending on different DTT concentrations were observed for TTR samples supplemented with 200 mM Ca^2+^ and incubated at room temperature for long durations (Fig. 1). Importantly, a separate sample was prepared for each incubation time and each DTT concentration. The emission spectrum of dbAF was observed on Day 1 in the TTR sample, which contained the highest concentration of DTT (40 mM) (Fig. 1A). TTR spectra obtained after longer incubation times in the presence of 40 mM DTT did not show dbAF. Because the presence of the highly unstable conformer 2, immediately preceding TTR precipitation, has been previously observed in SDS gels at 40 mM DTT, the precipitation of the subpopulation of dbAF exhibiting TTR molecules was very likely [34]. The formation of dbAF could also be observed on Day 3 in the TTR sample containing 20 mM DTT (Fig. 1B). Interestingly, the spectrum showed only one peak (at 410 nm) of the “typical” dbAF spectrum, which was observed for the TTR sample containing 20 mM DTT on Day 4 (Fig. 1C), and after a longer incubation time under these conditions, dbAF was not observed (Fig. 1D). The dbAF was recorded for the TTR sample, which contained the lowest DTT concentration (1 mM) after 4 days of incubation, but the dbAF intensity decreased after longer incubation (Fig. 1D). This experiment shows that the dynamics of the formation of dbAF emitters in TTR molecules are dependent on the redox potential of the environment. One cannot exclude the possibility that dbAF was formed between the incubation times that were chosen to record the dbAF spectra in TTR samples that contained other DTT concentrations.

### 3.2 dbAF and dityrosine signatures are present in the subpopulations of TTR and depend on redox conditions and Ca^2+^

**Fig. 2:**
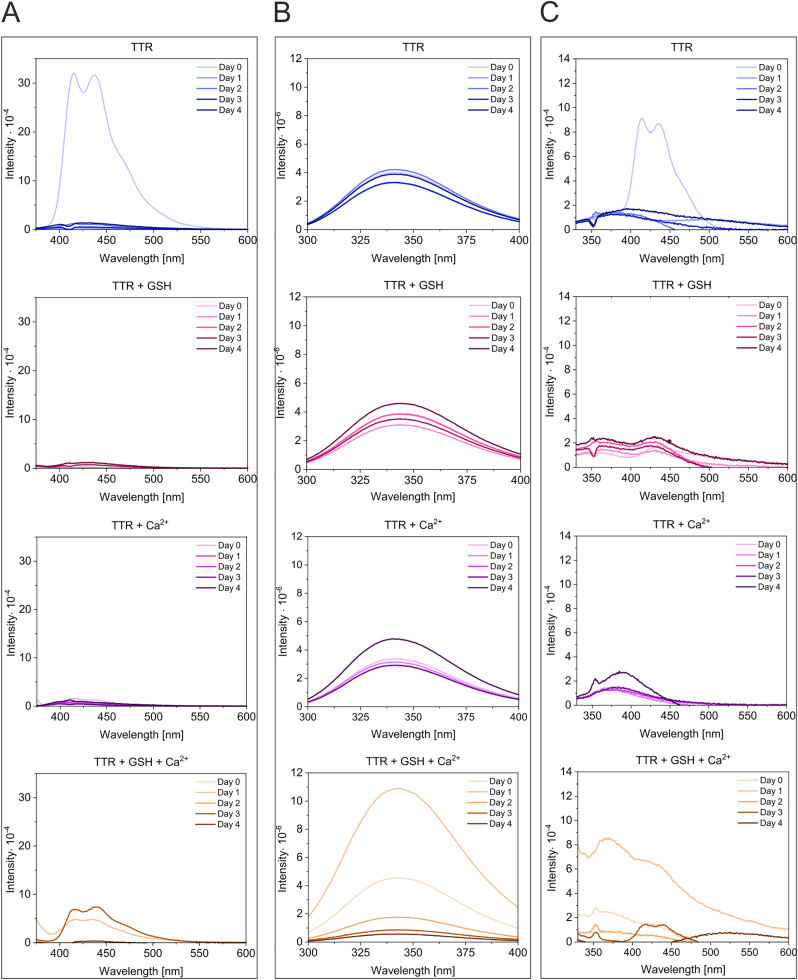
**GSH and Ca**^**2+**^**induce the formation of highly unstable forms of TTR** Samples of TTR (2.3 μM) in Tris buffer were supplemented as indicated with 100 mM GSH and 200 mM CaCl_2_ at final concentrations. Samples, prepared in quintuplicate, were incubated at room temperature, and the fluorescence emission spectra were recorded after the indicated periods of time for each sample. The spectra obtained using excitation of 360 nm (A), 275 nm (B) and 315 nm (C) at all incubation times for each sample were superimposed.

To be able to measure dbAF in identical samples but minimize the effect of irradiation-induced oxidation on dbAF fluorophore formation (as spectra recording is linked to the exposure of the sample to UV irradiation), each TTR sample was prepared in quintuplicate. TTR samples with and without 200 mM Ca^2+^ and/or 100 mM GSH, which is a physiological redox buffer, were incubated for different time periods, as shown in Fig. 2. After a given incubation time, three different spectra were recorded for each quintuplet sample: with excitation at 360 nm (dbAF) (Fig. 2A), 275 nm (aromatic fluorophores) (Fig. 2B) and 315 nm (Fig. 2C). The wavelength of 315 nm is one of the absorption maxima of dityrosine, as protein oxidation often results in dityrosine formation [[36], [37], [38]]. We monitored the fluorescence spectra of TTR using 315 nm excitation. The dbAF was observed for TTR samples incubated for the shortest time (Fig. 2A, Day 0). When this sample was excited at 315 nm, a spectrum analogous to dbAF but of lower intensity was observed (Fig. 2C, Day 0), indicating only that the dbAF fluorophore was present and excited outside of its maximum. In the TTR sample containing Ca^2+^ and GSH, the formation of dbAF was observed on Days 1 and 3 (Fig. 2A). Importantly, the formation of dbAF-emitting molecules on Day 1 was accompanied by a significant increase followed by a strong decrease in the intensity of the aromatic fluorophores (Fig. 2B). Such dramatic changes in the intensity of aromatic fluorophores may accompany structural rearrangements preceding precipitation and suggest the formation of a highly unstable (and dbAF-emitting) TTR subpopulation. Previously, we observed that dbAF formation was correlated with the cumulative TTR precipitation occurring in subpopulations over long time periods [34]. Please note that the dbAF observed in these conditions on Day 3 was not associated with a large change in the fluorescence of aromatic fluorophores. Except for the TTR sample, which contained both Ca^2+^ and GSH, the intensities of the aromatic fluorophores of the other analyzed samples did not change significantly regardless of the dbAF presence (Fig. 2B). Intriguingly, excitation of the sample containing Ca^2+^ and GSH with a wavelength of 315 nm resulted in a unique emission spectrum exhibiting maxima at approximately 365 nm and 406–407 nm observed on Day 1. These observations suggested that despite the GSH presence, dityrosine may be formed in a subpopulation of highly unstable TTR molecules (Fig. 2C, Day 1).

**Fig. 3:**
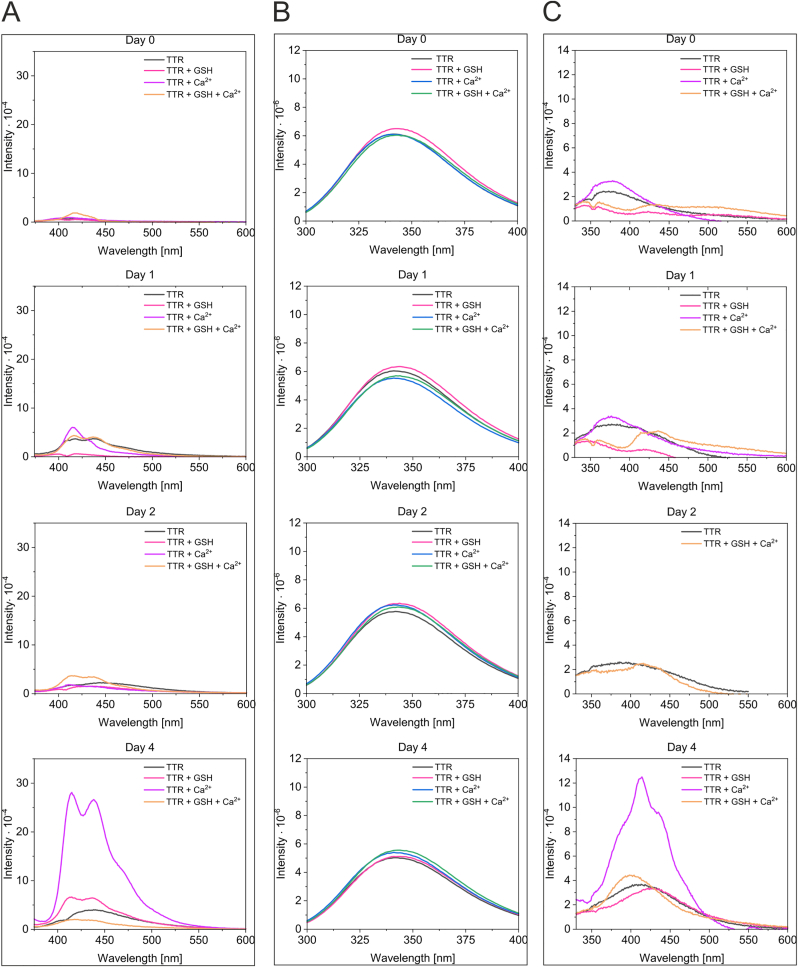
**GSH and Ca**^**2+**^**induce the formation of dbAF and dityrosine in substoichiometric populations of TTR** Samples of TTR (4.1 μM) in Tris buffer were supplemented as indicated with 2 mM GSH and 200 mM CaCl_2_ at final concentrations. Samples were incubated at room temperature, and for each sample, the fluorescence emission spectra were recorded after the indicated periods of time. The spectra obtained for all samples using excitation of 360 nm (A), 275 nm (B) and 315 nm (C) were superimposed for each incubation time. The same sample was used for spectra recording after each incubation.

Each TTR tetramer contains five tyrosine residues. Extended incubation time and/or irradiation of the sample during spectrum recording increases the likelihood of protein oxidation and photoactivated dityrosine formation. The residues of Tyr116 in the dimer-forming subunits (A-B and *C*-D) are located close enough to each other (3.9 Å) in the TTR structure to be able to form covalent bonds, resulting in crosslinked dimers, provided that this process is accompanied by conformational changes in TTR. To increase the exposure of TTR to oxidizing conditions, GSH was added to the samples at a lower concentration (2 mM) compared to the previous experiment, and the same sample was used to record spectra of dbAF (Fig. 3A, excitation at 360 nm), aromatic fluorophores (Fig. 3B, excitation at 275 nm), and dityrosine (Fig. 3C, excitation at 315 nm) after each incubation time. Unique dynamics of dbAF formation were observed for each sample (Fig. 3). The dbAF spectrum of the TTR sample containing both destabilizing factors (GSH and Ca^2+^) showed a single peak of very low intensity with a maximum at 417 nm on Day 0 (Fig. 3A). A typical dbAF spectrum of higher intensity was observed on Days 1 and 2, but it was not observed later (Day 4). In the TTR sample containing only Ca^2+^, one peak with a maximum at 415 nm was observed on Day 1, two peaks with maxima at 411 nm and 430–435 nm of very low intensity were emerging on Day 2, and a typical dbAF spectrum of high intensity was observed on Day 4. In the TTR sample containing only GSH, a typical dbAF spectrum was observed on Day 4. In the TTR sample, which was incubated in the absence of destabilizing agents, a dbAF spectrum was observed on Day 1, but it was transformed into a low intensity, broad spectra with maxima of 442–451 nm and 431–445 nm on Days 2 and 4, respectively. Importantly, the relative intensities of the aromatic fluorophores (excitation at 275 nm) for all analyzed samples did not change in parallel (Fig. 3B) with respect to the changes occurring in the corresponding dbAF spectra (Fig. 3A), confirming the formation of substoichiometric population of TTR molecules emitting dbAF.

Fluorescence emission spectra, obtained with excitation at 315 nm (Fig. 3C) for the TTR samples incubated in the absence of free thiols (TTR and TTR + Ca^2+^) and recorded after the shortest incubation times (Days 0 and 1), showed shallow emission maxima in the range of 370–380 nm. Increasing the incubation time (Day 2) resulted in a redshift of the maximum, suggesting changes occurring in the structures of TTR molecules. On Day 4, a shallow peak with a maximum at 413 nm and a strong heterogeneous peak with a maximum at 414 nm were observed for the TTR and TTR supplemented with Ca^2+,^ samples, respectively. Fig. S1 shows the superimpositions of the spectra of dbAF, aromatic fluorophores and dityrosine for each sample. Such alignment allows us to observe a decrease in the intensity of aromatic fluorophores occurring with increasing incubation time, caused by the cumulative precipitation of substoichiometric populations of unstable TTR molecules. Such arrangements of spectra also allow us to directly compare the modulatory effects of oxidizing conditions (multiple irradiation cycles of the same sample with lower GSH concentration used) in comparison to previous experiments (Fig. 2).

**Conclusion:** the presence of free thiols leads to frequent formation of dbAF-emitting forms of TTR that are highly unstable and prone to precipitation. The oxidative conditions to some extent counteract the destabilizing effects of GSH and Ca^2+^ and may lead to dityrosine formation. The dityrosine signature was observed in GSH-free samples (TTR and TTR + Ca^2+^).

### 3.3 The dbAF spectrum reflects TTR structural/oxidative changes

**Fig. 4:**
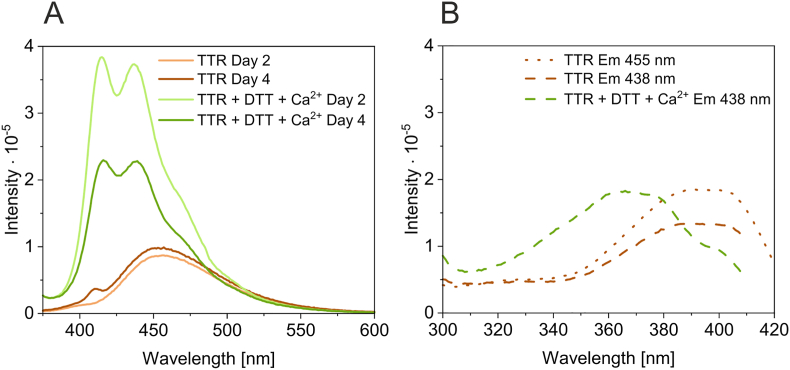
**Structural/oxidative changes in TTR are reflected in dbAF spectra** Samples of TTR with a *C*-terminal histidine tag (4.4 μM) in 30 mM Tris, 60 mM NaCl, pH 7.3 were subjected to a thermal cycle of denaturation (from 20 °C to 80 °C) and renaturation (from 80 °C to 20 °C) in the absence or presence of 200 mM CaCl_2_. Next, each sample was divided into two portions, and one half of each sample was supplemented with 1 mM DTT. All samples were incubated at room temperature, and the fluorescence emission spectra were recorded with excitation at 360 nm after 2 and 4 days of incubation (A). After the third day of incubation, the fresh portion of DTT (1 mM final concentration) was added to the sample that was previously supplemented with DTT. (B) shows the excitation spectra collected at 438 nm and 455 nm for the samples with corresponding emission spectra shown in (A).

Since we observed that small portions of unstable, dbAF-emitting TTR molecules were formed in our experimental system, the TTR molecules in the remaining pool were subjected to conditions that adversely affected their structure for long durations. To determine whether structural destabilization accelerates the process of dbAF formation, we tested TTR samples that had been previously subjected to destabilizing (challenging) conditions. Therefore, to record fluorescence emission spectra, we used TTR samples that were subjected to a cycle of thermal denaturation (from 20 °C to 80 °C) and renaturation (from 80 °C to 20 °C) prior to incubation with reducing agent (DTT). Prolonged incubation of such structurally challenged TTR in the presence of DTT and Ca^2+^ resulted in the formation of dynamic subpopulations emitting strong dbAF (Fig. 4A). Spectra recorded for TTR samples that were not exposed to reducing conditions and Ca^2+^ showed the emission of dbAF exhibiting only one peak with a maximum of 456–457 nm. A similar spectrum was previously observed for TTR sample that was subjected to multiple irradiation cycles/oxidizing conditions (Day 2 in Fig. 3A). The excitation spectra of challenged TTR subjected to different redox conditions also showed significant differences (Fig. 4B). The excitation spectrum of the TTR sample supplemented with both destabilizing factors, DTT and Ca^2+^, had a broad maximum in the range of 360–380 nm and corresponded to/was identical to the excitation spectrum observed previously [34], whereas the excitation spectrum obtained for the TTR sample without Ca^2+^ and DTT showed a broader maximum in the range of 379–406 nm. Therefore, we concluded that TTR that was exposed to oxidizing conditions and TTR that was exposed to free thiols and/or Ca^2+^ must be structurally different and/or must undergo different modifications. Because these forms of TTR exhibited dbAF with different properties, the oxidation/structural state of TTR is reflected in the spectroscopic and fluorescence properties of dbAF.

### 3.4 Riboflavin-enhanced photooxidation is reflected in the fluorescent properties of TTR

**Fig. 5:**
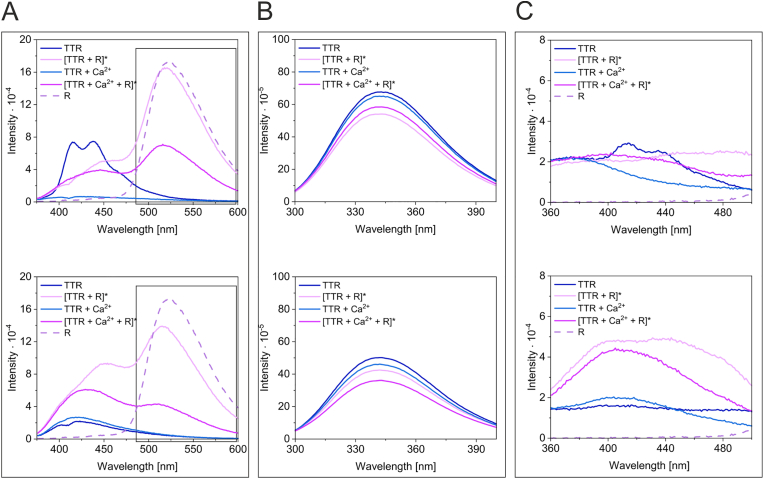
**Riboflavin-sensitized oxidation of TTR leads to unique dbAF and dityrosine spectra and indicates riboflavin binding to TTR** Samples of TTR (5.2 μM) in HEPES buffer were supplemented with 30 mM riboflavin and/or 200 mM CaCl_2_ (final concentrations) as indicated. Samples were irradiated at 23 °C for 30 min using an excitation wavelength of 445 nm and slits of 2.0 nm. All samples were then applied to G-25 spin columns and centrifuged. Fluorescence emission spectra were recorded with excitation at 360 nm (A), 275 nm (B) and 315 nm (C) immediately (upper panels) and after incubation overnight at 60 °C (lower panels). The samples that were subjected to irradiation are marked with asterisks. The rectangle indicates the fluorescence spectrum of riboflavin.

Multiple irradiation cycles, prolonged incubation or other conditions affecting the structural stability may result in protein oxidation and/or contribute to dbAF and dityrosine (or another covalent crosslink) formation [39]. To confirm the correlation between oxidation state and TTR properties, we subjected TTR samples (in the presence and absence of Ca^2+^) to irradiation using riboflavin as a sensitizer. This experimental system should reflect the oxidation process of TTR occurring in vivo. The samples were irradiated for 30 min using an excitation wavelength of 445 nm. Control samples of TTR and TTR supplemented with Ca^2+^ were not irradiated or exposed to riboflavin but were incubated at room temperature for analogous periods of time. Then, all samples were applied to G-25 spin columns and centrifuged. This protocol should result in the separation of riboflavin from TTR in riboflavin-containing samples. Spectra of dbAF (Fig. 5A), aromatic fluorophores (Fig. 5B), and dityrosine (Fig. 5C) were recorded immediately (upper panels) and after overnight incubation of all samples at 60 °C (Fig. 5, lower panels). The dbAF was observed only for the TTR sample that was not irradiated (TTR sample, Fig. 5A top panel). The TTR samples that underwent irradiation in the presence of riboflavin (denoted in Fig. 5 as [TTR + R]* and [TTR + Ca^2+^ + R]*) showed two peaks: one peak with a maximum at approximately 442–462 nm and a second peak (with a maximum at approximately 516–523 nm). The second peak (Fig. 5A, rectangle) closely overlapped with the riboflavin spectrum; therefore, we concluded that these TTR samples must still contain some riboflavin. The amount of riboflavin remaining in the sample could only be estimated; therefore, precise correction of the riboflavin contribution to the TTR spectrum (and precise determination of the location of the first maximum of TTR emission) was not possible. Overnight incubation of the samples at 60 °C resulted in the precipitation of a subpopulation of dbAF-emitting molecules (Fig. 5A, compare top panel with bottom panel), which was reflected in the decrease in the intensity of aromatic fluorophores and the absence of dbAF in the TTR sample (Fig. 5B). However, the decrease in fluorescence intensity of aromatic fluorophores was most pronounced for riboflavin-containing irradiated TTR sample supplemented with Ca^2+^. This indicated that the destabilizing factors (irradiation, riboflavin, Ca^2+^, and high temperature) had a synergistic effect on TTR stability. Excitation at 315 nm (Fig. 5C, top panel) reflected the presence of dbAF in the TTR sample and produced a shallow peak at 371–374 nm in the TTR sample containing Ca^2+^. The irradiated samples did not indicate dityrosine emission immediately after irradiation, but after overnight incubation at 60 °C (Fig. 5C, lower panel), the emission spectra exhibited maxima at approximately 404–410 nm in both TTR samples that were irradiated in the presence of riboflavin (Fig. 5C, lower panel, samples [TTR + R]* and [TTR + Ca^2+^ + R]*). A similar peak, but of much lower intensity, was also observed in the TTR sample that was not irradiated but contained Ca^2+^. The spectrum of the [TTR + R]* sample contained a second overlapping peak with a maximum at approximately 438–439 nm. Contribution of riboflavin emission in this range (although not very pronounced) to the fluorescence of dityrosine may be responsible for this peak.

Collectively, these data indicate that in the absence of free thiols and/or due to prolonged incubation and/or light exposure of TTR in the presence of riboflavin, dityrosine may be formed in TTR as a result of oxidation. Furthermore, dbAF spectra of TTR samples that were irradiated in the presence of riboflavin showed a unique maximum located approximately at 450 nm, which resembled the dbAF spectrum observed for challenged TTR (Fig. 4). Because riboflavin emission was observed in the samples (despite the separation procedure), we concluded that riboflavin or riboflavin photoproducts bind to TTR under our experimental conditions.

### 3.5 Riboflavin and/or riboflavin photoproducts bind to TTR

To verify the putative binding of riboflavin or riboflavin photoproducts, TTR samples that had been irradiated in the presence of riboflavin and nonirradiated TTR samples were subjected to analytical gel filtration using a Superdex S75 Increase column (Fig. S2). Elution profiles were monitored for protein at 280 nm (black) and for riboflavin at 440 nm (red). TTR tetramers eluted close to the void volume. The TTR peak overlapped with the low intensity peak of riboflavin (indicated with the arrow in Fig. S2), as seen in the elution profiles of the samples of TTR and TTR supplemented with Ca^2+^ that were irradiated in the presence of riboflavin. The peak of riboflavin also overlapped with the TTR tetramer in the sample that was preincubated with riboflavin after TTR irradiation and in the TTR sample that was preincubated with riboflavin but not irradiated. In the first sample, the riboflavin peak had very low intensity, and in the latter sample, two subpopulations of riboflavin-bound TTR molecules were present. In irradiated samples, peaks of very low intensities at 280 nm were also observed in the void volume (Figs. S2B and S2C, asterisks), suggesting that irradiation decreased TTR stability and led to the formation of substoichiometric populations of oligomers. Destabilization of TTR was particularly evident in samples that were incubated overnight at 60 °C after irradiation (Fig. S2C). The maximum riboflavin absorption lies in the range of 310–700 nm, and the absorbance at 440 nm has been used for riboflavin quantification [40]. However, riboflavin also absorbs light at 280 nm, as seen in the elution profiles of riboflavin alone and the profiles of TTR samples containing riboflavin (see the peaks of an exclusion volume (Vi) in Fig. S2). In the elution profiles of TTR samples that were irradiated in the presence of riboflavin, the peaks in the Vi volume were heterogeneous and therefore should represent riboflavin photooxidation products (Fig. S2, rectangles). The size-exclusion chromatography experiment confirmed that TTR binds riboflavin and/or riboflavin photoproducts and indicated that irradiation leads to destabilization and oligomerization of TTR.

#### 3.5.1 Riboflavin-enhanced photooxidation affects the stability and molecular properties of TTR

TTR destabilization is linked to tetramer dissociation and monomer unfolding. To test which process is affected by oxidation, the relative unfolding rate of TTR irradiated in the presence and absence of riboflavin was determined using the S-Trap assay [14,41]. The R_ui_ parameter was determined using SDS–PAGE and densitometric analysis as described previously [41]. The R_ui_ parameter reflects the unfolding rate (the relative amount of monomer) observed after heating the TTR samples in the presence of SDS at 85 °C for different time periods (0 s–1200 s). Fig. S3 shows that riboflavin-enhanced photooxidation of TTR significantly increased the rate of TTR unfolding. As Ca^2+^ also increased TTR unfolding, this experiment confirmed the destabilizing action of both agents. Surprisingly, TTR photooxidation in the absence of these factors seems to have negligible effect on TTR unfolding, indicating that the mechanisms of oxidation-induced destabilization are significantly modulated by Ca^2+^ and riboflavin.

**Fig. 6:**
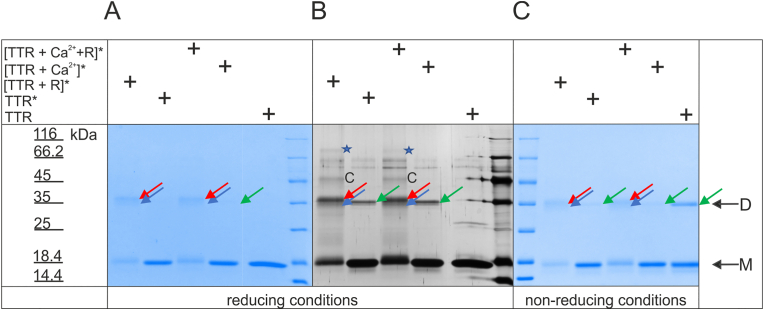
**An anomalous TTR dimer resistant to dissociation under reducing conditions is formed under riboflavin-sensitized oxidation** Samples of TTR (8.7 μM) in HEPES buffer were supplemented with 100 μM riboflavin and/or 100 mM CaCl_2_ (final concentrations) as indicated and were irradiated at 23 °C for 30 min using an excitation wavelength of 445 nm and slits of 2.0 nm. Then, the samples were incubated overnight at 60 °C, and 50 μg (100 μL) of each sample was applied to a Superdex S75 Increase column. Thirty microliters of TTR peak fraction of each sample eluted from the column with Tris buffer was mixed with four times concentrated sample buffer supplemented with (A and B) and devoid of (C) 2.5% β-mercaptoethanol. Then, the samples were subjected to heat denaturation at 95 °C for 20 min (A and B) or at 95 °C for 15 min, except for the untreated TTR sample, which was denatured at 85 °C for 30 min (C). The gels were run in a Laemmli buffer system and stained with Coomassie Brilliant Blue R250 (A and C) and silver (B). The arrows indicate anomalous dimer (red), dimer (green) and truncated dimer (blue). C and blue asterisks indicate the conformer and the oligomeric forms of TTR, respectively. (For interpretation of the references to colour in this figure legend, the reader is referred to the Web version of this article.)

When TTR samples irradiated in the presence of riboflavin were analyzed using SDS–PAGE, in addition to the monomer band (M), dimers (D) of TTR with abnormal mobility (indicated by red arrows) were observed after staining the gels with Coomassie (Fig. 6A and C) or silver (Fig. 6B). Additionally, riboflavin-enhanced irradiation of TTR led to the presence of forms of much lower mobility (indicated by C and asterisks). These forms corresponded to oligomers in the elution profiles of equivalent samples analyzed by size-exclusion chromatography (Fig. S2). Prolonged denaturation of the sample under reducing conditions (Fig. 6A and B) did not result in the dissociation of the anomalous dimeric form of TTR or forms exhibiting lower mobility. The TTR samples that were irradiated in the absence of riboflavin contained only a negligible amount of the dimeric form of TTR (Fig. 6, green arrows), and both forms (dimeric and monomeric) in these samples exhibited mobility consistent with that of untreated TTR, as seen in the gels obtained using reducing conditions (Fig. 6B) and during milder denaturation under nonreducing conditions (Fig. 6C). In the TTR samples that were irradiated in the presence of riboflavin, the mobility of the monomeric and dimeric forms was slightly lower than that of the corresponding bands for the TTR sample that was not irradiated or for the samples that were irradiated in the absence of riboflavin. This observation again suggests covalent binding of riboflavin or riboflavin photoproducts to TTR. Interestingly, dimers of slightly higher mobility (blue arrows) were observed in all irradiated TTR samples, which may reflect TTR proteolysis.

**Fig. 7:**
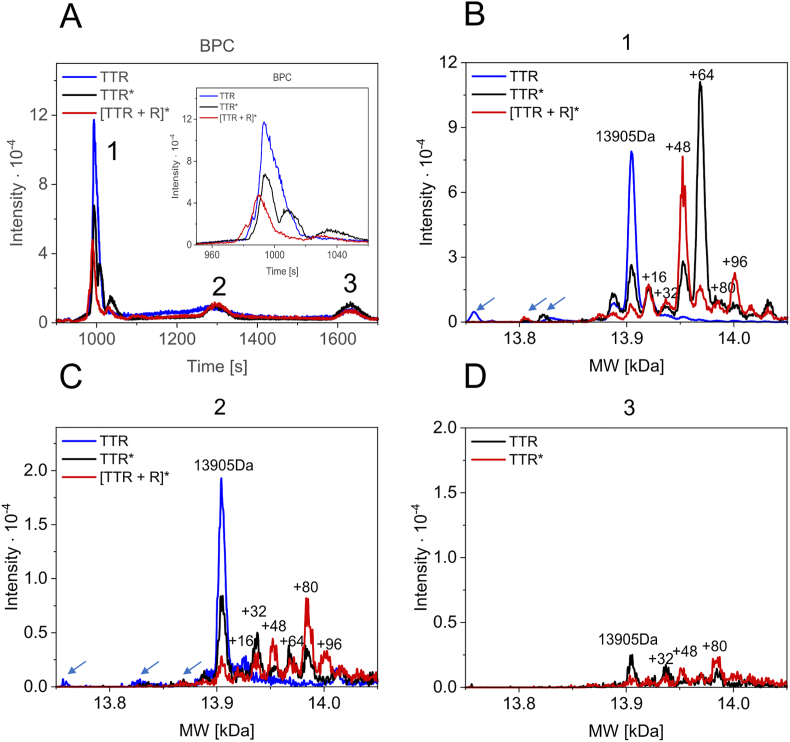
**Irradiation results in the formation of multiple oxidized forms of TTR** TTR samples (35 μM monomer) in HEPES buffer were preincubated in the absence or presence of riboflavin (100 μM) and subjected to irradiation at 23 °C for 30 min using an excitation wavelength of 445 nm and 2.0 nm slits. The irradiated samples were incubated overnight at 60 °C and subjected to size-exclusion chromatography on a Superdex S75 Increase column. The peak protein fractions and nonirradiated TTR (50 μM monomer) in HEPES buffer were vacuum dried, resuspended in 2% acetonitrile with 0.05% TFA and centrifuged at 21,000×*g* for 15 min at 4 °C. Fifty nanograms of each protein sample was separated on a 15 cm × 75 μm Accucore™ 150-C4 column (A). MS spectra were obtained for the main protein peaks. The MW determination of the protein by a deconvolution multiply charged ion series was performed using maximum entropy software. (B, C, D) The superimpositions of the protein deconvoluted spectra obtained for peaks 1, 2 and 3 of all samples. The insert in A shows the enlargement of superimposed peak no. 1.

To verify the results of TTR irradiation at the molecular level, samples of TTR photooxidized in the absence and presence of riboflavin and samples of TTR that were not irradiated were subjected to reversed-phase chromatography using an Accucore™ column. Base peak chromatograms (BPC) showed three populations of TTR molecules in each sample (1, 2 and 3, Fig. 7A). The predominant population of molecules eluted in peak 1. Irradiation resulted in significant changes in the properties of TTR, as population 1 molecules in the nonirradiated TTR sample eluted as one main asymmetric peak, consisting of multiple subpopulations with closely overlapping retention times (Fig. 7A, insert). In contrast, population 1 in photooxidized TTR samples (TTR* and [TTR + R]*) eluted in at least three peaks with different retention times. Each of three populations (1–3) of irradiated TTR samples and two populations (1 and 2) of nonirradiated TTR samples, all separated via reversed-phase chromatography, were subjected to MS. Superimpositions of the deconvoluted spectra obtained for population 1 and population 2 for nonirradiated and irradiated TTR samples are shown in Fig. 7B and C, respectively. Superimposition of the spectra obtained for population 3 of irradiated TTR samples is shown in Fig. 7D. The nonirradiated TTR contained mostly unmodified TTR monomers with MWs equal to 13,905 Da (ProtParam). In the irradiated samples, the amounts of unmodified TTR monomers were much lower (Fig. 7B and C). In population 1 (Fig. 7B) of nonirradiated TTR, only two forms with MWs shifted by −17 Da and +16 Da were observed, whereas numerous modified/oxidized forms of TTR were observed in high amounts in the photooxidized TTR samples. The differences in the MWs of the photooxidized TTR molecules were multiples of 16 Da, which clearly indicates the consecutive addition of oxygen atoms. The most abundant oxidized form of TTR irradiated in the absence of riboflavin was 13,969.5 Da (+64.5 Da), whereas riboflavin-enhanced photooxidation resulted in a dominant form of 13,952.2 Da (+47.2 Da) TTR (Fig. 7C). Populations 2 and 3 (Fig. 7C and D) of the irradiated samples also contained predominantly modified/oxidized forms of TTR, but the total amount of protein in these populations was much lower than that in population 1 (please note the scale differences in Fig. 7B compared to Fig. 7C and D). The distributions of oxidized forms of TTR between populations 1–3 were unique. Interestingly, in population 2 of TTR molecules irradiated in the absence of riboflavin, the peak corresponding to the unmodified monomer was relatively high (Fig. 7C) in comparison to population 1 (Fig. 7B). Additionally, the differences in patterns of monomer truncations between nonirradiated and irradiated samples (Fig. 7B and C, arrows) were sample- and population-specific.

When spectra obtained for population 1 after deconvolution were compared over a wider range of MWs (from 12,500 Da to 20,000 Da, Fig. S4), it was clearly visible that in all samples, the MWs of most TTR molecules were centered around the MWs of the TTR monomer and its oxidized forms. However, two symmetrically distributed populations of TTR molecules were observed (Fig. S4, rectangles): one with MWs lower by hundreds of Da (MWs in the range of 12,500 to 13,500) and the other with MWs higher (MWs in the range of 14,500 Da to 15,500 Da) than the MWs of unmodified or oxidized forms of the TTR monomer. Populations within the lower MW range represent truncated TTR monomers. The second population, which contains molecules with MWs exceeding the MW of the TTR monomer, must correspond to monomers that had been covalently bound to short fragments of the other TTR monomer. We previously observed and named this population “monomer plus molecules” [41]. Significantly increased quantities of truncated forms were observed in the irradiated TTR samples in comparison to the nonirradiated samples (Fig. S4, compare A, B to C, D). However, for monomer plus molecules, no concomitant, irradiation-induced increase was observed that would correspond to an increase in the quantity of truncated forms of TTR. Instead, TTR molecules with MWs exceeding the MW of the TTR monomer throughout the whole range were more abundant than in the nonirradiated samples. In addition, only in the irradiated samples were molecules with MW of approximately 18,700 Da observed (Fig. S4, asterisks). This MW corresponds to a subpopulation of TTR monomers covalently linked to relatively long fragments (approximately 4800 Da) of another TTR monomer. The pairwise superimposed MS spectra before deconvolution, obtained for population 1 of irradiated and nonirradiated TTR samples (Fig. S5), showed that irradiation reduced the quantity of forms with a lower *m*/*z* ratio (Fig. S5C and Fig. S5D), suggesting a stronger compaction of these molecules. One exception was the ions of low *m*/*z* values indicated with the asterisks in Figs. S5C and D, which were present in the irradiated TTR samples. The inserts in Fig. S5 C and D show the detailed spectra of these ions. Three ions with *m*/*z* values of 546.7 (1091 Da), 547.7 (1093 Da) and 555.7 (1123 Da) were present in TTR samples irradiated in the absence of riboflavin, with the latter being dominant. In the TTR sample irradiated in the presence of riboflavin, only one ion of *m*/*z* equal to 547.6 (1093 Da) was observed. The MW (indicated in parenthesis) distribution suggests that these ions represent short, approximately ten-amino-acid-long peptides that are differentially modified. The MW difference between the major ions is equal to 30 Da, which may represent di-oxidation of a peptide occurring concomitantly with double deprotonation in the TTR sample irradiated in the absence of riboflavin. The minor ions of *m*/*z* 547.7 (1093 Da) and 546.7 (1091 Da) in the TTR sample irradiated in the absence of riboflavin might represent unmodified and doubly deprotonated forms of the same peptide, respectively.

**In conclusion**, irradiation of TTR decreases its stability and leads to oligomer formation and oxidation of TTR. Riboflavin and/or photoproducts of riboflavin bind to TTR and lead to TTR dityrosine formation and/or crosslinking. Importantly, irradiation-induced oxidation increases TTR proteolysis, leading to the formation of two types of truncated forms of TTR molecules; TTR devoid of short peptides (with approximately 10 amino acid residues), and TTR fragments formed by cleavage located closer to the center of the TTR molecule. Importantly, the oxidation and structural states of TTR are reflected in the dynamics and unique dbAF spectra.

## 4 Discussion

TTR is susceptible to oxidative modifications, and multiple forms of TTR were found to be correlated with the redox state of the environment [31]. Each TTR monomer possesses one cysteine residue (Cys10) located at the end of its flexible N-terminus. The thiol group of Cys10 is particularly susceptible to modifications that change its redox state [31]. It can be fully reduced (-SH), can form disulfide bridges with cysteine, homocysteine, glycine, cysteinylglycine, glutathione and other compounds [42], can be linked to sulfenic (-SOH), sulfinic (-SO_2_H) or sulfonic (-SO_3_H) acid groups or can be mono-, di- or tri-oxidized to the oxide or acid form [29,31]. Accordingly, many different Cys10-modified TTR isoforms were found in plasma and in CSF [31,32,42]. The modification of Cys10 of TTR has physiological consequences. For example, plasmatic TTR differentially modified at Cys10 exerted substantially different effects on Aβ aggregation and toxicity than unmodified recombinant TTR [43]. It has been shown that TTR Cys10 di-oxidation to sulfinic acid (+32 Da) accelerated the aggregation of Aβ peptide but suppressed Aβ-induced neurotoxicity [43]. Di-oxidation occurred only in the absence of free thiols such as DTT or GSH, and the addition of these compounds during the purification procedure eliminated di-oxidation [43].

The oxidation state of Cys10 strongly affects the structure of TTR; therefore, Cys10 can be regarded as a sensor of the redox state of the environment [30]. However, there are apparently conflicting data on the influence of the Cys10 oxidation state on the amyloid-forming propensity of TTR. On the one hand, some data show that the stability of TTR is enhanced in the oxidizing environment. Under mild acidic conditions, the modification of TTR with mixed disulfides, which is often observed in body fluids, particularly with cysteine but also with cysteinylglycine and glutathione, negatively influenced TTR stability, whereas sulfonation (-SO_3_H adduction) inhibited TTR from acid-induced fibrillation [44]. It has also been found that TTR, which is S-sulfonated and S-cysteinylated (plasmatic TTR), is more stable against acid denaturation than the unmodified or di-oxidized form of TTR (recombinant TTR) [43]. The structural basis for sulfite-induced stabilization of TTR was resolved by obtaining the crystal structure of sulfonated TTR [45]. Two of the three oxygen atoms provide stabilizing interactions with TTR amino acid residues His56, Gly57 and indirectly Thr59 (via water molecule), which are located in the DE loop. These interactions prevent displacement of strands C and D, which would promote amyloid formation [45]. Additionally, the oxidation of Met13 has been found to inhibit the fibril formation propensity of TTR [46]. On the other hand, other reports show that TTR stability is compromised by a highly oxidizing environment. It has been shown that S-sulfonation (adduction of –SO_3_H) decreases the stability of TTR, and sulfonation was proposed to be the triggering factor for fibril formation [47]. TTR molecules that were oxidized at Met13 and Cys10 residues, representing the oxidative modifications related to aging (Met13 oxide, Cys10 sulfonic acid and carbonylation), were less stable and more cytotoxic than wild-type TTR [29]. Both Met13 and Cys10 have been found to be oxidized in the fibrillar form of TTR [48]. Our data may shed some light on these seemingly contradictory observations. We confirm the sensitivity of TTR to the redox state of the environment, and we show that the structure of TTR is unstable outside of a narrow range of redox conditions. This is rational because TTR, as an oxidative stress sensing factor, should be sensitive to both strongly reducing and strongly oxidizing conditions.

The dbAF emitting molecules constitute very small portions (substoichiometric populations) of the analyzed TTR samples. This correlated with our earlier observations that highly unstable, prone to precipitation, substoichiometric populations of TTR were formed in the presence of high concentrations of Ca^2+^ [41]. Importantly, dbAF dynamics were dependent on the balance between reductive and oxidizing conditions (redox balance) of the environment. Moreover, in our experimental system, free thiols (DTT or physiological redox agent, GSH) counteracted the exposure of TTR samples to oxygen during prolonged incubation. However, both reducing and oxidizing factors affected the TTR structure and led to the appearance/formation of intrinsic dbAF emitters, which exhibited unique and divergent excitation and emission spectra. The formation of dbAF emitters occurred in parallel with TTR dynamic structural changes, which led to oligomerization and subsequent cumulative precipitation. Therefore, the monitoring (following and documentation) of the dynamics of the dbAF phenomenon was very challenging.

The mechanism of dbAF phenomenon is not yet fully understood and it may not be straightforward as dbAF, in addition to proteins, was documented also for other polymers and simple organic compounds [49,50]. The dbAF of proteins seems to occur mainly due to the emission of excited electrons of peptide bonds in the hyperstable β-sheet structure [51,52]. Recent studies have shown that other structure-related factors such as non-covalent π-π stacking between tyrosine residues, chirality of n→π* transitions or supramolecular packing contribute to the spectroscopic properties of the dbAF fluorophores [49,50,52]. The ordered arrangement of atoms and the organization of hydrogen bonds cause a lowering of the energy barrier of electron transitions of the fluorophore, resulting in the emission of light in the visible range [[51], [52], [53]]. It has been found that dbAF occurs in structurally diverse proteins such as structured proteins and those containing intrinsically disordered regions of significant length [34,49,51,[53], [54], [55]]. Although the mechanism of excitation of the dbAF fluorophore(s) may be similar (or the same), the process of formation of the dbAF-emitting fluorophore(s) should be unique for different proteins, because they have different compositions of intrinsic fluorophores and also because their aggregation pathways are different. Therefore, dbAF can be used to monitor structural rearrangements of proteins in general, and the aggregation process in particular [34,55]. In addition, the formation of dbAF-emitting fluorophore(s) may be affected by factors that modify/affect the protein structure [56]. Our report confirms such hypothesis.

The fact that dbAF as well as MS spectra of nonoxidized and photooxidized (irradiated) TTR forms are different (in peak locations and intensities) and the observation of enhanced proteolysis of TTR in irradiated samples correlates with the in vivo observation of two types of TTR amyloid [27]. Amyloid B-type fibers have been observed in heart tissue sections of FAP patients. These fibers were highly congophilic and contained full-length TTR. Amyloid of A type was found in SSA and some FAP patients. It is weakly congophilic and is composed of full-length molecules and fragments of TTR [27]. In addition to the short N-terminal truncations, proteolytic fragmentation, produced by the cleavage of the peptide bond around the amino acid 48 residue, was found to be present in type A TTR amyloid deposits in vivo [27]. When the samples of amyloid A and B were analyzed by SDS–PAGE, with and without reduction and alkylation, some of the bands (5 kDa and 15 kDa) almost disappeared [27], indicating that the fragment and monomer of TTR were held together via disulfide bonds. These observations are consistent with our MS data. First, in all analyzed TTR samples, the peaks corresponding to the truncated (monomer minus) and TTR fragments covalently bound to other TTR monomer (monomer plus) subpopulations were observed in the deconvoluted MS spectra. Enhanced truncation of TTR in the presence of Ca^2+^ has been reported previously [41]. However, photooxidation resulted in a stronger increase in the quantity of the fragments compared to the effect exerted by Ca^2+^. Moreover, species of MW of approximately 18,700 Da, detected in the photooxidized TTR samples, may correspond to the full-length (or N-terminally truncated) monomer and the amyloid-associated longer proteolytic fragment, of similar MW to the MW of the fragment found in vivo in amyloid A-type, which are held together via covalent bond(s). Additionally, photooxidation of TTR resulted in the presence of ions of low *m*/*z* (equal to 546.7, 543.7 and 555.7) in the MS spectra. The ion spectral distributions let us assume that these ions represent differently modified peptide that is approximately ten amino acids long (1093 Da) and is present in TTR samples irradiated in the presence and absence of riboflavin. In TTR samples irradiated in the absence of riboflavin, this peptide is either oxidized (1123 Da) or doubly deprotonated (1091 Da). Intriguingly, the ions have the same charge (+2). It is tempting to speculate that electron-coupled deprotonation of tyrosine 114 and 116 residues with the formation of a tyrosyl radical and/or oxidation of these tyrosine residues occurs. However, these ions may represent the fragmentation of the N-terminus of TTR and the oxidation of Cys10 or Met13 residues.

Cys10 sulfonation (+80 Da) of TTR has been observed in SSA and FAP patients and was correlated with fibril formation propensity [47]. Accordingly, we observed elevated levels of +80 Da modification of TTR in the photooxidized samples. The occurrence of +80 Da and +96 Da oxidative modifications in photooxidized TTR suggests that S-sulfonation and oxidation of the S atom of Cys10 to sulfonic acid may occur, which would only be possible with disulfide bridge formation followed by sulfur oxidation at one of the Cys10 residues. These observations are consistent with the fact that amyloid deposits (especially in patients with SSA) are linked to oxidation, which is associated with aging (oxidative stress). Accordingly, our data show that photooxidized TTR was prone to oligomerization and precipitation.

Plasma treatment with hypochlorous acid resulted in TTR and other protein crosslinking and dityrosine formation [57]. Dityrosine is a marker of oxidative stress. Our data show that under the experimental conditions of induced oxidative stress (riboflavin-sensitized photooxidation), tyrosyl radical and dityrosine formation occur in TTR. The fluorescence emission at 404–410 nm after the excitation at 315 nm and the crosslinking of the irradiated TTR monomers observed in SDS–PAGE would indicate dityrosine formation. The dityrosine emission spectrum with a maximum in the 400–409 nm range has been found previously for other proteins [37,58]. The electron photoejection and formation of tyrosyl radicals have been observed after irradiation of tyrosine in water solutions [59]. It has also been observed that such tyrosyl radical formation results in fluorescence emission from 400 to 440 nm [60]. Accordingly, the second emission peak with an apparent maximum of 438–439 nm was recorded in the TTR samples that were irradiated in the presence of riboflavin. The effectiveness of dityrosine formation in UV-irradiated solutions is low (below 6%) [58]. Therefore, strong pro-oxidizing conditions, such as multiple irradiations and prolonged incubation, thermal denaturation or sensitization with riboflavin, were required to induce the fluorescence emission, which could be attributed to dityrosine. Although we were unable to observe the absorption/excitation of dityrosine at 315 nm, we instead observed strong absorption at 275 nm, suggesting that dityrosine is protonated/unionized [37,58]. In SDS–PAGE, we observed in riboflavin-sensitized photooxidized samples of TTR the dimer of abnormal (reduced) mobility, which was insensitive to reducing agent treatment. The mobility of the monomer was also slightly decreased. This was the indirect confirmation of the binding of riboflavin or riboflavin photoproducts to TTR and/or crosslinking of TTR molecules. Riboflavin is a potent antioxidant in the dark and is also a component of FMN and FAD nucleotides, which are the coenzymes of dehydrogenases and oxidases [61]. Under UV light exposure, intermolecular electron rearrangements result in riboflavin photoproducts, and riboflavin can generate ROS such as superoxide anions and singlet oxygen [61].

Many oxidized forms of TTR were found in the most abundant population 1 of the irradiated TTR molecules when analyzed in MS. The increment in the MWs of the nonnative forms in the irradiated TTR samples was equal to +16 Da, suggesting addition of the subsequent oxygen atoms. The MW of the most prevalent form of the irradiated TTR was shifted by +64 Da, whereas riboflavin-sensitized TTR photooxidation resulted in the domination of the +47.2 Da form of TTR, and the form of +96 Da was also enhanced. Intriguingly, the most abundant form of TTR oxidation in the presence of riboflavin bears one fewer oxygen atom than the oxidized TTR form obtained in the absence of riboflavin. Therefore, riboflavin either protects TTR against oxidation at one site or changes the oxidation pattern. Both effects indirectly indicate the binding of riboflavin to TTR. Previous reports have shown that Cys10 and Met13 are primary oxidized amino acid residues in TTR. Cys10 was found to be oxidized to sulfenic, sulfinic and sulfonic acids, which resulted in +16 Da, +32 Da and +47.2 Da shifts, respectively [31,43,48]. Met13 was found to be oxidized to sulfoxide and di-sulfoxide with +16 Da and +32 Da shifts in MW, respectively [46]. However, the tyrosine and tryptophan residues are also very susceptible to photooxidation in proteins, especially in the presence of riboflavin [39], and the oxidized TTR form displaying higher shifts (+80 Da and +96 Da) may represent the oxidation of these amino acids or other oxidative modifications at multiple amino acid residues in addition to Cys10 S-sulfonation. UV irradiation of ubiquitin (and Aβ and insulin) led to the oxidative modification of tyrosine residues, resulting in aggregation and formation of the fluorophore emitting blue light in the range of 350 nm–550 nm. The maximum fluorescence emission of UV-oxidized ubiquitin was ca. 450 nm, which correlates with our finding for TTR [39]. The UV-induced oxidative changes in ubiquitin were attributed to the formation of dityrosine and DOPA [39].

Importantly, our observations may shed some light on the pathogenesis of oxidation/ROS-induced diseases such as ocular tissue diseases, systemic TTR amyloidosis and multiple sclerosis. The interphotoreceptor matrix (IPM) is localized in the eye between the retina and RPE*.* The RPE is the source of TTR and RBP, and the presence of both proteins in the IPM has been unequivocally confirmed [2]. Riboflavin and other flavins are present at high concentrations in ocular tissues [62]. Although the protein retbindin binds riboflavin and other flavins and protects ocular tissues from light-induced damage, it is mainly localized to the RPE microvilli and photoreceptor interface [62]. Therefore, TTR can be exposed to riboflavin and UV light in the IPM. This may, at least partially, contribute to the pathophysiology of TTR amyloid depositions in the eye.

We observed that the influences of anti- (free thiols) and pro-oxidizing (irradiation, long incubation) conditions on TTR structure and stability were antagonistic, but the effects of Ca^2+^ and free thiols or Ca^2+^ and pro-oxidizing factors were synergistic. In particular, Ca^2+^ augmented the effect of GSH on dbAF emission. These and our previous data showed that TTR stability was compromised by Ca^2+^ at high concentrations [41], but imbalanced redox conditions also led to TTR amyloid formation. High concentrations of free thiols and riboflavin-sensitized photooxidation of TTR resulted in a stronger effect than Ca^2+^ and both factors (oxidation and high Ca^2+^ concentration) contribute to amyloid deposition. It has been recently found that TTR amyloid deposits of type A (composed of the full length and truncated TTR forms) in the heart tissue sections of patients with systemic TTR amyloidosis were accompanied by Ca^2+^ depositions of unique characteristics and unusual morphology [63]. These dispersed, unevenly distributed Ca^2+^ depositions had a cloud-like appearance and contained very small mineralo-organic particles. The presence of membranous structures and positive ubiquitin and p62 staining indicated that the cellular process (possibly autophagy) underlies the pathogenesis of Ca^2+^ deposition. The presence of iron indicated the involvement of the mitochondria. Therefore, it has been proposed that defective autophagy is responsible for the formation of TTR amyloid. Interestingly, localizations of TTR amyloid deposits were only partially colocalized with calcifications [63]. Defective autophagy/mitophagy that would result in ROS and Ca^2+^ leakage from defective mitochondria would compromise the stability of TTR and lead to TTR amyloid and Ca^2+^ deposition. The negative and synergistic influences of both ROS and Ca^2+^ on TTR stability would explain the partial colocalization of amyloid and microcalcifications. TTR was found to interact with mineral nanoparticles [64], and the link between TTR aggregation and the process of mineralization has been previously reported [41,65].

The forms of TTR with lower mobilities in SDS–PAGE indicated the presence of aggregated and oxidized TTR forms, and TTR conformers were observed in the irradiated TTR samples. In the CSF of multiple sclerosis patients [66], TTR oxidized forms and anomalous dimers, which are susceptible to dissociation in reducing conditions [33], were observed and were specifically associated with the disease [33]. Multiple sclerosis is a demyelinating inflammatory disease of the central nervous system [67]. The multiple modifications of TTR molecules specifically associated with multiple sclerosis may be markers of imbalanced redox conditions in the brain. Concomitantly, the modified/oxidized and crosslinked TTR molecules may actively contribute to the pathogenesis of multiple sclerosis, either due to the lack of native TTR function or due to the switch of TTR from a protective [23] to a pathological component of the redox vicious cycle.

## Declaration of competing interest

The authors declare no conflict of interest.

## Data Availability

Data will be made available on request.
