# Supplementary material for: Deep blue autofluorescence reflects the oxidation state of human transthyretin
Source: Redox Biol. 2022 Aug 9;56:102434. doi: 10.1016/j.redox.2022.102434 (PMC9411673; doi:10.1016/j.redox.2022.102434)
Supplement: Multimedia component 1 [file mmc1.pdf]

Supplementary Data

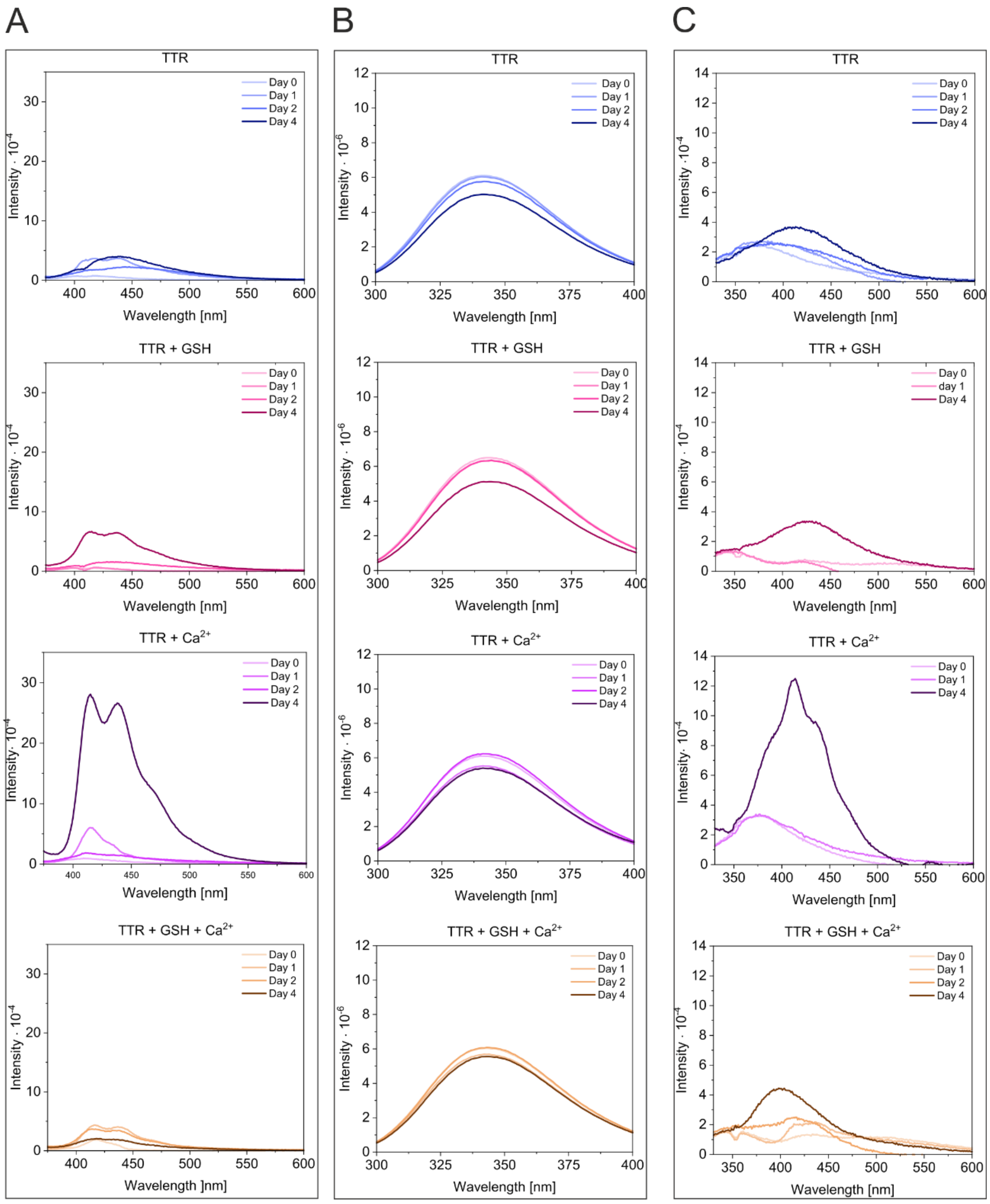

Figure S1

GSH and Ca^{2+} induce the formation of dbAF and dityrosine in substoichiometric populations of TTR

Samples of TTR (4.1 µM) in Tris buffer were supplemented as indicated with 2 mM GSH and 200 mM $CaCl_2$ at final concentrations. Samples were incubated at room temperature, and for each sample, the fluorescence emission spectra were recorded after the indicated periods of time. The spectra obtained using excitation of 360 nm (A), 275 nm (B) and 315 nm (C) at all incubation times for each sample were superimposed. The same sample was used for spectra recording after each incubation.

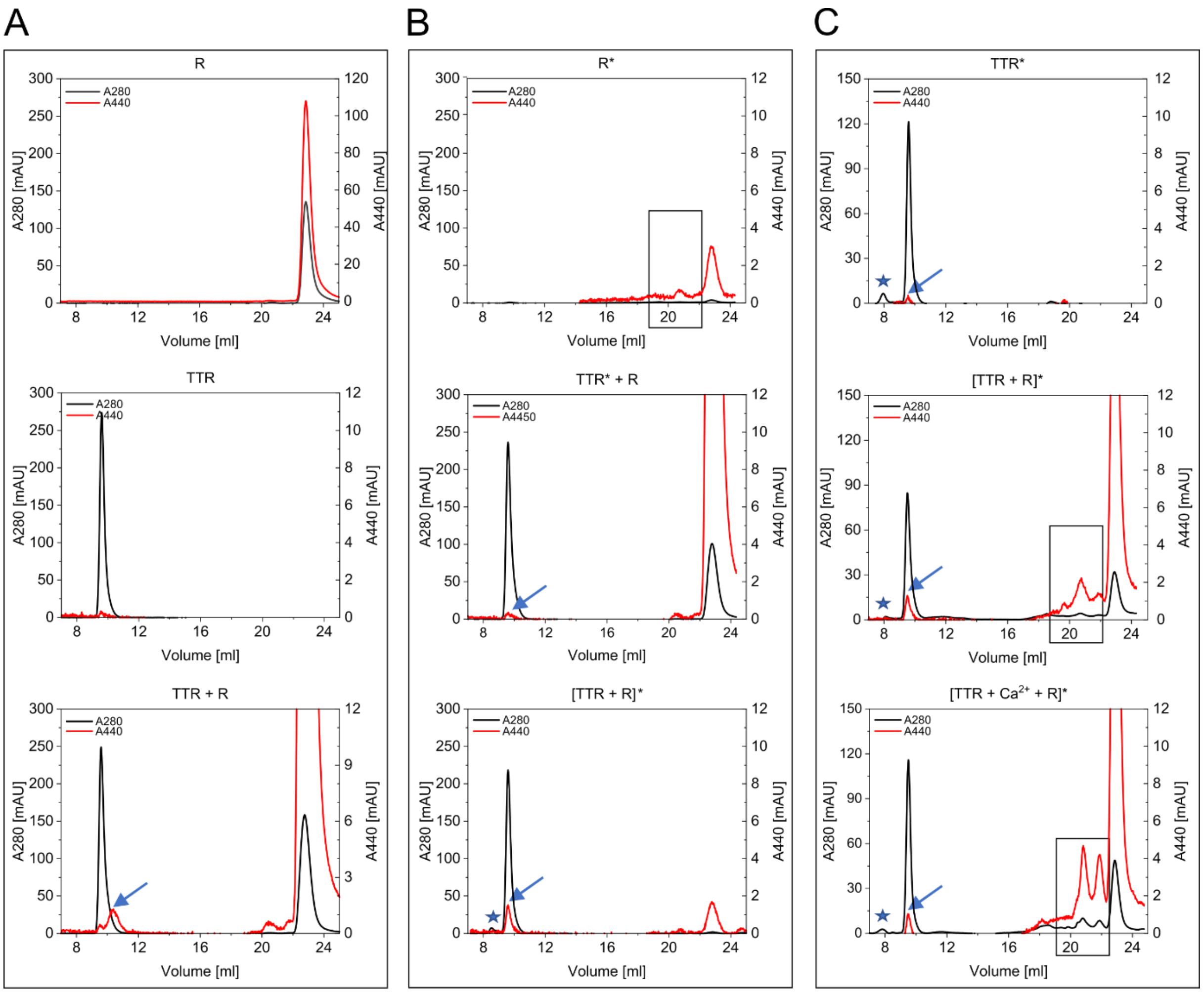

Figure S2

TTR binds riboflavin and/or riboflavin photoproducts

(A, B) Samples of TTR (35 µM) in Tris buffer were supplemented with 350 µM riboflavin and irradiated at 23 °C for 30 minutes using an excitation wavelength of 445 nm and slits of 2.0 nm, as indicated with the asterisk in the sample name. One hundred microliters (100 µg) of each sample was applied to a Superdex S75 Increase column and eluted with Tris buffer. (C) Samples of TTR (3.5 µM 8.7 µM) in HEPES buffer were supplemented with 30 µM 100 µM riboflavin and/or 200 mM 100 mM $CaCl_2$ (final concentrations) as indicated. Samples were irradiated at 23 °C for 30 minutes using an excitation wavelength of 445 nm and slits of 2.0 nm and were incubated overnight at 60 °C. One hundred microliters (50 µg) of each sample was applied to a Superdex S75 Increase column and eluted with Tris buffer. The absorption at 280 nm and 440 nm was measured to monitor protein and riboflavin, respectively. The asterisks indicate TTR oligomers, and arrows indicate riboflavin bound by TTR tetramers. Rectangles indicate the products of the photooxidation of riboflavin.

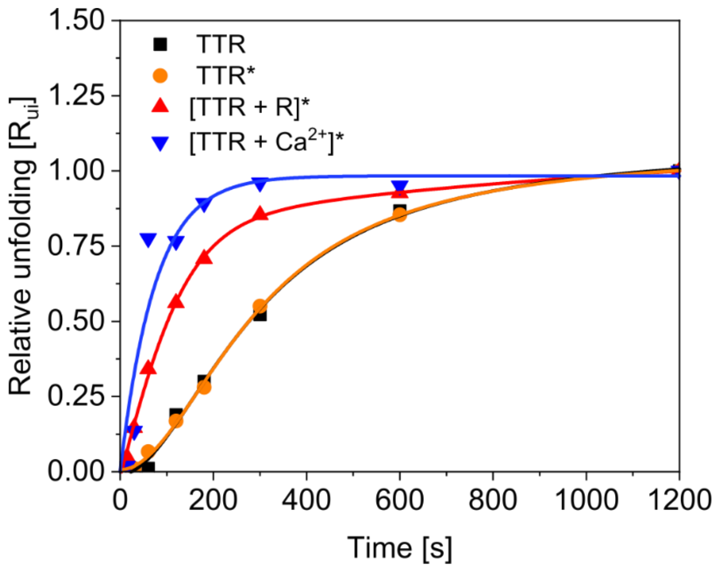

Figure S3

Riboflavin-sensitized oxidation increases the unfolding rate of TTR

Samples of TTR (35 µM) in Tris buffer were supplemented with 350 µM riboflavin and/or 200 mM $CaCl_2$ and were either nonirradiated or irradiated at 23 °C for 30 minutes using an excitation wavelength of 445 nm and slits of 2.0 nm as indicated. Then, for each TTR sample, the S-Trap experiment was performed: the TTR samples (9 µg) were subjected to unfolding for 0, 15, 30, 60, 120, 180, 300, 600 and 1,200 s at 85 °C after the addition of 5 times concentrated SDS-buffer preheated to 95 °C. Next, the samples were cooled on ice, spun and separated using 12% SDS gels and stained with Coomassie. After destaining, the intensities of the bands corresponding to the monomer and dimer were determined for each gel. The intensity of the dimer band at 0 s of unfolding was set as the standard of quantity. The relative intensity of the monomer after time *i* of incubation was determined by calculating the R_{ui} parameter equal to $(U_i-U_0)/(U_{final}-U_0)$, where U_i, U_0 and U_{final} are the intensities of the monomer bands at given times of 0 s and 1,200 s, respectively. The irradiated samples are indicated by asterisks.

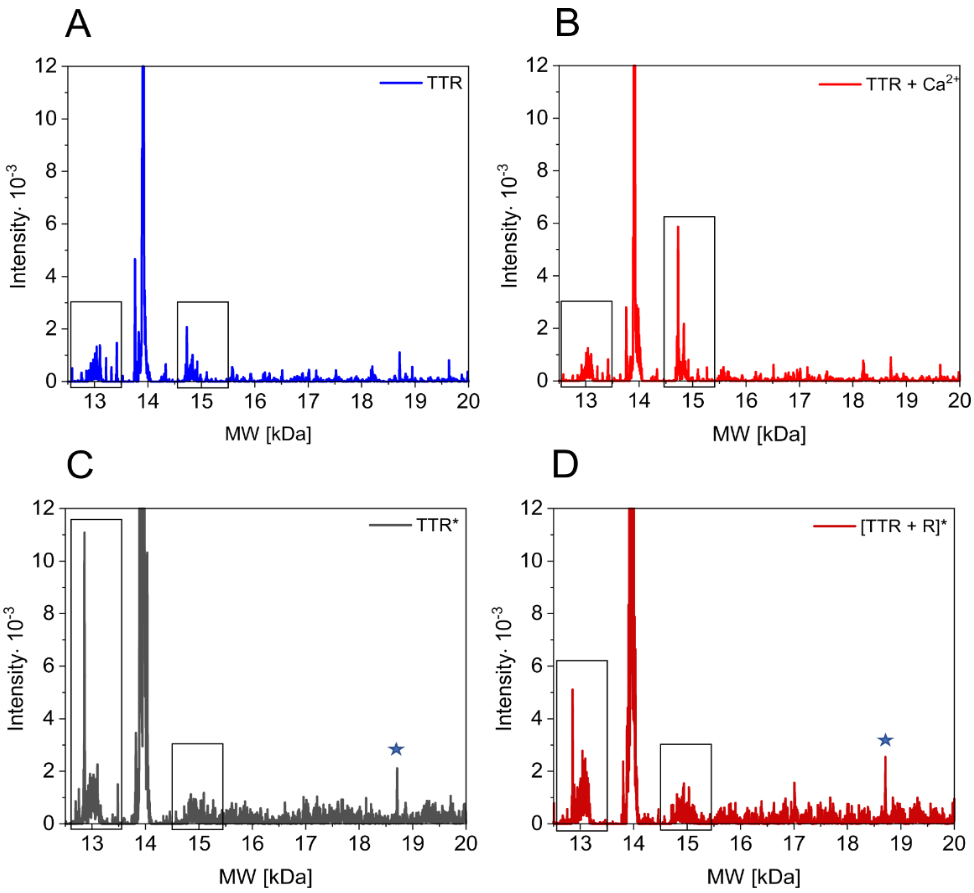

Figure S4

Irradiation enhances TTR degradation

TTR samples (35 µM monomer) in HEPES buffer were preincubated in the absence or presence of riboflavin (100 µM) and were subjected to irradiation at 23 °C for 30 min using an excitation wavelength of 445 nm and 2.0 nm slits. The irradiated samples were incubated overnight at 60 °C and subjected to size-exclusion chromatography on a Superdex S75 Increase column. The peak protein fractions and nonirradiated TTR samples (50 µM monomer) in HEPES buffer (which were preincubated in the absence and presence of 200 mM $CaCl_2$) were vacuum dried and resuspended in 2% acetonitrile with 0.05% TFA and centrifuged at 21,000 × g for 15 min at 4 °C. Fifty nanograms of each protein sample was separated on a 15 cm×75 µm Accucore™ 150-C4 column. MS spectra were obtained for the main three protein peaks corresponding to three populations (1-3) of TTR molecules separated via reversed-phase chromatography. (A-D) The MW spectra as determined by a deconvoluted multiply charged ion series for population 1 of all TTR samples using maximum entropy software. Rectangles indicate the monomer-minus and monomer-plus ranges of MWs. The asterisks indicate the subpopulations with MWs of ca. 18,700 Da.

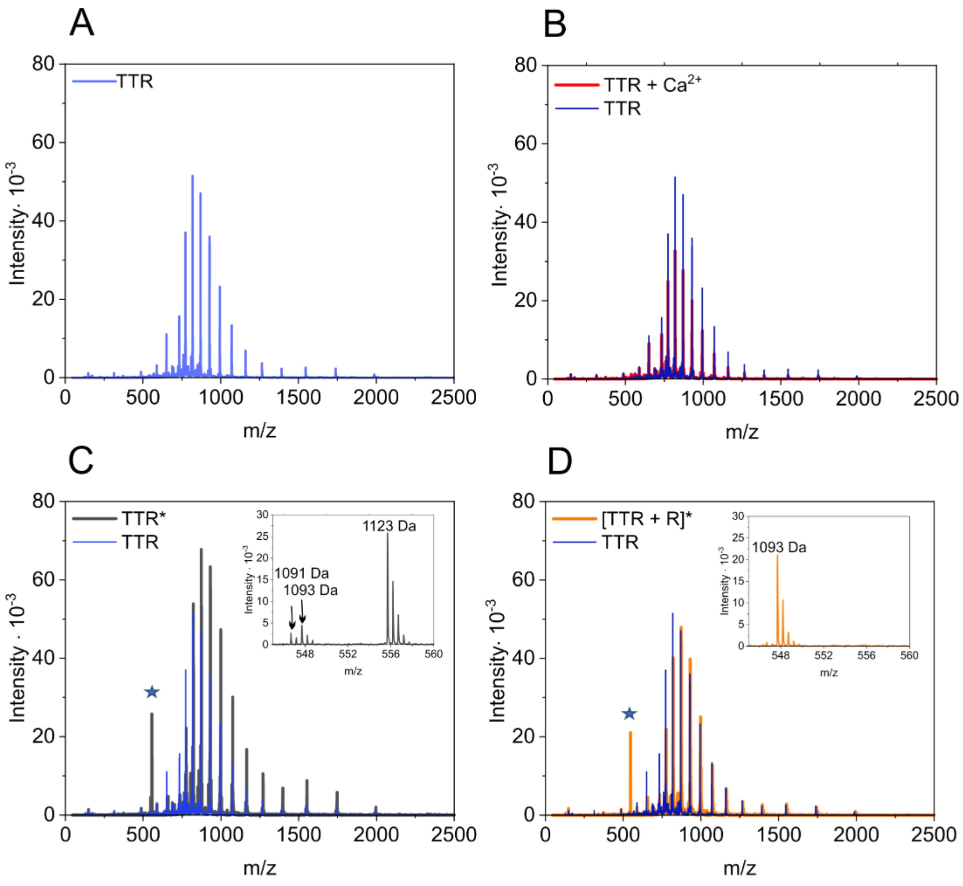

Figure S5

Irradiation affects the structure (m/z ratio) of TTR molecules

TTR samples (35 µM monomer) in HEPES buffer were preincubated in the absence or presence of riboflavin (100 µM) and subjected to irradiation at 23 °C for 30 min using an excitation wavelength of 445 nm and 2.0 nm slits. The irradiated samples were incubated overnight at 60 °C and subjected to size-exclusion chromatography on a Superdex S75 Increase column. The peak protein fractions and nonirradiated TTR samples (50 µM monomer) in HEPES buffer (which were preincubated in the absence and presence of 200 mM $CaCl_2$) were vacuum dried and resuspended in 2% acetonitrile with 0.05% TFA and centrifuged at 21,000 × g for 15 min at 4 °C. Fifty nanograms of each protein sample was separated into three populations (1-3) of molecules on a 15 cm×75 µm Accucore™ 150-C4 column, and MS spectra were obtained for all populations of TTR from all samples. (A) MS spectrum for nonirradiated TTR. The pairwise superimpositions of the spectra of population 1 of TTR and TTR preincubated in the presence of $CaCl_2$ (B), TTR irradiated in the absence of riboflavin (C) and TTR irradiated in the presence of riboflavin (D). The inserts show the detailed spectra of ions (marked with asterisks) with m/z of 555.7 and 547.7, which are present in the samples TTR* and [TTR + R]*, respectively.
